# Abundance and recruitment data for *Undaria pinnatifida* in Brest harbour, France: Model versus field results

**DOI:** 10.1016/j.dib.2016.02.075

**Published:** 2016-03-04

**Authors:** James T. Murphy, Marie Voisin, Mark Johnson, Frédérique Viard

**Affiliations:** a Sorbonne Universités, UPMC Univ Paris 6, CNRS, UMR 7144, Adaptation & Diversity in Marine Environment Department, Divco Team, Station Biologique de Roscoff, Place Georges Teissier, 29680 Roscoff, France; b Marine Environment Research Group, Ryan Institute, National University of Ireland Galway, Galway, Ireland

**Keywords:** Macroalgae, Individual-based model, Invasive species, Seaweed, Kelp, Undaria pinnatifida, Recruitment, Abundance

## Abstract

The data presented in this article are related to the research article entitled “A modelling approach to explore the critical environmental parameters influencing the growth and establishment of the invasive seaweed Undaria pinnatifida in Europe” [1]. This article describes raw simulation data output from a novel individual-based model of the invasive kelp species *Undaria pinnatifida*. It also includes field data of monthly abundance and recruitment values for a population of invasive *U. pinnatifida* (in Brest harbour, France) that were used to validate the model. The raw model output and field data are made publicly available in order to enable critical analysis of the model predictions and to inform future modelling efforts of the study species.

**Specifications table**

**Table:** 

Subject area	*Biology*
More specific subject area	*Computational modelling of invasive macroalgae*
Type of data	*Tables*
How data was acquired	*Field survey, Individual-based model*
Data format	*Raw*
Experimental features	*Field data:* 64 aluminium panels set-up one metre below the water surface attached to pontoons in harbor setting.
Data source location	*Brest harbor, Brittany, France.*
Data accessibility	*Data is available with this article*

**Value of the data**

• This data facilitates the data collection of other researchers attempting to follow the same technique or to evaluate future methods for analysis of the data.
• There are limited public datasets available on the monthly abundance/recruitment of field populations of *U. pinnatifida* despite their importance for understanding invasion dynamics.
• Environmental parameters included so that the quantitative relationship between the population dynamics and environmental factors can be explored.
• Allows researchers to independently verify the model predictions versus field results.

## 1 Data

Table 1, Table 2a, Table 2b, Table 3a, Table 3b, Table 4 display raw model output and field data for populations of *Undaria pinnatifida* growing in a harbour setting. Model results are from simulations carried out using a spatially-explicit, individual-based model of *U. pinnatifida* population dynamics. A description of this model can be found in the associated research article [1]. Field data are from populations of invasive *U. pinnatifida* growing in Brest harbour, France, which were surveyed during 2005 and 2006 [2].

## 2 Experimental design, materials and methods

Field data was collected from the port of Brest in France during the 2005/06 growing season: during this field experiment, 64 aluminium panels were set-up one metre below the surface, a depth optimal for the recruitment of the *U.pinnatifida*, and the settlement and length of each individual was recorded every month.

Simulations were carried out using an individual-based model with environmental parameters (light, temperature and day length) representative of Brest harbour, France. Surface water temperature data for the port of Brest (2003–06) were obtained from a SOMLIT (Service d’Observation en Milieu Littoral, INSU-CNRS, Brest) buoy situated a few hundred metres from the marina [2], [3]. Mean global solar irradiance data for the region were obtained using the CalSol online application (Institut National de L׳Energie Solaire, CEA-CNRS) [4].

## Figures and Tables

**Table 1:** Raw model output from simulation of *Undaria pinnatifida* population. Abund=No. of sporophyte agents; Recruit=No. of new sporophyte agents (<1 month old); Gameto=No. of gametophyte agents; Spores=total no. of spores in the environment; Temp=water temperature (°C); Solar=Solar radiation (Megajoules m^−^^2^ h^−^^1^); D.L.=day length (day light hours).

**Month**	**Abund**	**Recruit**	**Gameto**	**Spores**	**Temp**	**Solar**	**D.L.**
**1**	0	0	4000	0	9.73	0.38	8.89
**2**	0	0	3704	0	9.22	0.65	10.17
**3**	40	40	3263	0	9.75	0.92	12.06
**4**	192	178	2943	0	11.12	1.12	13.95
**5**	171	84	3249	9.3E+09	13.15	1.24	15.39
**6**	66	15	10349	3.7E+10	15.35	1.27	16.01
**7**	13	12	12702	2.2E+09	16.89	1.24	15.62
**8**	16	13	12440	2.6E+09	17.54	1.12	14.30
**9**	78	75	11986	2.8E+09	17.05	0.93	12.47
**10**	190	167	11884	8.4E+09	15.59	0.67	10.58
**11**	287	221	11704	4.9E+09	13.46	0.40	9.09
**12**	279	151	10746	6.6E+08	11.39	0.27	8.49
**13**	319	153	9569	1.7E+03	9.78	0.38	8.89
**14**	565	359	8358	0	9.18	0.65	10.17
**15**	762	445	7329	0	9.68	0.92	12.06
**16**	859	421	6660	0	11.20	1.12	13.95
**17**	745	184	9933	7.5E+10	13.20	1.24	15.39
**18**	282	34	44969	1.8E+11	15.25	1.27	16.01
**19**	26	22	50902	5.2E+09	16.85	1.24	15.62
**20**	78	75	48893	2.9E+09	17.43	1.12	14.30
**21**	204	189	47027	1.3E+10	16.93	0.93	12.47
**22**	595	545	44873	2.0E+10	15.47	0.67	10.58
**23**	1092	861	43183	2.7E+10	13.46	0.40	9.09
**24**	1103	607	40653	2.8E+09	11.33	0.27	8.49
**25**	1212	568	36309	7.6E+08	9.83	0.38	8.89
**26**	2063	1281	31735	0	9.15	0.65	10.17
**27**	2896	1691	27677	0	9.67	0.92	12.06
**28**	3362	1591	25387	6.0E+09	11.20	1.12	13.95
**29**	2790	603	36802	2.9E+11	13.26	1.24	15.39
**30**	1046	114	153281	6.7E+11	15.38	1.27	16.01
**31**	109	94	171091	1.8E+10	16.94	1.24	15.62
**32**	238	217	164369	2.4E+10	17.42	1.12	14.30
**33**	830	774	156976	4.2E+10	16.96	0.93	12.47
**34**	2208	1979	149316	7.5E+10	15.51	0.67	10.58
**35**	3530	2759	143625	7.3E+10	13.35	0.40	9.09
**36**	3621	1996	132164	1.1E+10	11.31	0.27	8.49
**37**	4197	2045	117755	6.0E+08	9.73	0.38	8.89
**38**	7019	4300	103070	0	9.22	0.65	10.17
**39**	9309	5237	90333	6.6E+08	9.76	0.92	12.06
**40**	10645	4952	82258	1.6E+10	11.19	1.12	13.95
**41**	9138	2114	110277	9.4E+11	13.26	1.24	15.39
**42**	3537	449	421117	2.3E+12	15.40	1.27	16.01
**43**	302	234	473819	6.7E+10	16.89	1.24	15.62
**44**	657	605	453465	6.0E+10	17.41	1.12	14.30
**45**	2085	1917	429927	1.3E+11	16.88	0.93	12.47
**46**	5924	5332	405506	2.1E+11	15.50	0.67	10.58
**47**	9681	7642	383861	2.2E+11	13.47	0.40	9.09
**48**	9653	5375	353109	3.3E+10	11.35	0.27	8.49
**49**	11272	5569	313331	6.8E+06	9.77	0.38	8.89
**50**	19119	11902	274492	6.1E+08	9.15	0.65	10.17
**51**	25300	14090	240896	4.4E+05	9.66	0.92	12.06
**52**	28837	13260	219687	4.0E+10	11.18	1.12	13.95
**53**	24528	5480	285372	2.7E+12	13.17	1.24	15.39
**54**	9328	1166	956971	6.0E+12	15.26	1.27	16.01
**55**	846	653	1065121	2.0E+11	16.92	1.24	15.62
**56**	1649	1512	1016441	1.6E+11	17.44	1.12	14.30
**57**	4820	4449	967828	3.0E+11	16.94	0.93	12.47
**58**	13337	11938	903433	5.2E+11	15.43	0.67	10.58

**Table 2a:** Field data: Raw monthly abundance data for *Undaria pinnatifida* from Brest harbour, France 2005/06 [2], along with normalised values (expressed relative to peak in annual abundance). Letters a–e represent five different sets of colour-coded aluminium panels (*n*=64) installed in Brest harbour, attached to floating pontoons at a depth of 1 m below the water surface.

**Month**	**Raw data (Sporophytes m**^**−2**^**)**					**Normalised**					**Mean**	**SEM**
**a**	**b**	**c**	**d**	**e**	**a**	**b**	**c**	**d**	**e**
**Aug 05**	0.00	1.89	1.89	5.48	0.00	0.00	0.03	0.05	0.14	0.00	0.04	0.026
**Sep 05**	0.00	13.26	0.00	0.00	1.10	0.00	0.19	0.00	0.00	0.03	0.04	0.037
**Oct 05**	0.00	11.36	7.58	1.10	5.48	0.00	0.16	0.21	0.03	0.16	0.11	0.041
**Nov 05**	13.89	26.52	11.36	1.10	7.68	0.44	0.38	0.32	0.03	0.22	0.28	0.072
**Dec 05**	4.17	13.26	22.73	14.25	6.58	0.13	0.19	0.63	0.36	0.19	0.30	0.091
**Jan 06**	9.72	17.05	13.26	19.74	6.58	0.31	0.24	0.37	0.50	0.19	0.32	0.054
**Feb 06**	25.00	37.88	13.26	28.51	9.87	0.80	0.54	0.37	0.72	0.28	0.54	0.099
**Mar 06**	26.79	70.08	18.94	39.35	34.72	0.86	1.00	0.53	1.00	1.00	0.88	0.092
**Apr 06**	31.25	35.98	35.98	25.46	24.31	1.00	0.51	1.00	0.65	0.70	0.77	0.098
**May 06**	11.22	13.26	15.15	14.71	11.03	0.36	0.19	0.42	0.37	0.32	0.33	0.039
**Jun 06**	4.81	3.79	11.36	9.80	8.58	0.15	0.05	0.32	0.25	0.25	0.20	0.045
**Jul 06**	1.60	0.00	2.08	2.45	0.00	0.05	0.00	0.06	0.06	0.00	0.03	0.014

**Table 2b:** Model Output: Predicted sporophyte abundance. Raw data along with normalised values (expressed relative to peak in annual recruitment). Data from four simulated growth seasons (a–d).

**Month**	**Raw data (Sporophytes m**^**−2**^**)**				**Normalised**				**Mean**	**SEM**
**a**	**b**	**c**	**d**	**a**	**b**	**c**	**d**
**Aug 05**	6	36	214	518	0.02	0.02	0.03	0.02	0.02	0.003
**Sep 05**	23	138	689	2335	0.07	0.08	0.10	0.09	0.09	0.006
**Oct 05**	75	345	1815	5622	0.23	0.20	0.25	0.22	0.23	0.010
**Nov 05**	123	579	2561	7959	0.37	0.34	0.36	0.32	0.35	0.012
**Dec 05**	120	538	2332	7544	0.36	0.32	0.32	0.30	0.33	0.013
**Jan 06**	136	632	2431	7814	0.41	0.37	0.34	0.31	0.36	0.022
**Feb 06**	259	1231	4738	14176	0.79	0.73	0.66	0.57	0.69	0.048
**Mar 06**	316	1476	6245	22518	0.96	0.87	0.87	0.90	0.90	0.021
**Apr 06**	329	1689	7183	25040	1.00	1.00	1.00	1.00	1.00	0.000
**May 06**	283	1354	6067	19012	0.86	0.80	0.84	0.76	0.82	0.023
**Jun 06**	116	481	3480	5464	0.35	0.28	0.48	0.22	0.34	0.057
**Jul 06**	13	75	225		0.04	0.04	0.03		0.04	0.004

**Table 3a:** Field data: Raw monthly recruitment values for *Undaria pinnatifida* from Brest harbour, France 2005/06 [2], along with normalised values (expressed relative to peak in annual recruitment). Letters (a-e) represent five different sets of colour-coded aluminium panels (*n*=64) installed in Brest harbour, attached to floating pontoons at a depth of 1 m below the water surface.

**Month**	**Raw Data (Recruits m^−2^)**					**Normalised**					**Mean**	**SEM**
**a**	**b**	**c**	**d**	**e**	**a**	**b**	**c**	**d**	**e**
**Aug 05**	0.00	1.89	1.89	4.39	0.00	0.00	0.06	0.10	0.22	0.00	0.08	0.041
**Sep 05**	0.00	9.47	0.00	0.00	1.10	0.00	0.29	0.00	0.00	0.07	0.07	0.057
**Oct 05**	0.00	9.47	3.79	1.10	5.48	0.00	0.29	0.20	0.06	0.36	0.18	0.069
**Nov 05**	2.78	11.36	5.68	1.10	4.39	0.11	0.35	0.30	0.06	0.29	0.22	0.058
**Dec 05**	1.39	5.68	3.79	5.48	3.29	0.05	0.18	0.20	0.28	0.22	0.19	0.037
**Jan 06**	1.39	11.36	1.89	5.48	5.48	0.05	0.35	0.10	0.28	0.36	0.23	0.064
**Feb 06**	6.94	11.36	3.79	15.35	6.58	0.27	0.35	0.20	0.78	0.44	0.41	0.101
**Mar 06**	17.86	32.20	5.68	16.20	15.05	0.71	1.00	0.30	0.82	1.00	0.77	0.129
**Apr 06**	25.30	20.83	18.94	19.68	9.26	1.00	0.65	1.00	1.00	0.62	0.85	0.090
**May 06**	6.41	7.58	7.58	7.35	7.35	0.25	0.24	0.40	0.37	0.49	0.35	0.047
**Jun 06**	1.60	1.89	3.79	2.45	4.90	0.06	0.06	0.20	0.12	0.33	0.15	0.050
**Jul 06**	0.00	0.00	2.08	0.00	0.00	0.00	0.00	0.11	0.00	0.00	0.02	0.022

**Table 3b:** Model Output: Predicted number of recruits (sporophyte agent >5 cm in length and<1 month old). Raw data and normalised values (expressed relative to peak in annual recruitment). Data from four simulated growth seasons (a–d).

**Month**	**Raw data (Recruits m^−2^)**				**Normalised**				**Mean**	**SEM**
**a**	**b**	**c**	**d**	**a**	**b**	**c**	**d**
**Aug 05**	19	52	211	476	0.09	0.05	0.05	0.03	0.05	0.011
**Sep 05**	33	156	741	2130	0.15	0.15	0.18	0.15	0.16	0.008
**Oct 05**	71	381	1765	4819	0.32	0.36	0.43	0.34	0.36	0.023
**Nov 05**	112	556	2164	6062	0.51	0.52	0.52	0.42	0.49	0.023
**Dec 05**	85	416	1371	4496	0.38	0.39	0.33	0.32	0.36	0.019
**Jan 06**	76	395	1275	3907	0.34	0.37	0.31	0.27	0.32	0.021
**Feb 06**	220	975	3394	9262	1.00	0.91	0.82	0.65	0.84	0.074
**Mar 06**	221	1068	3916	14265	1.00	1.00	0.95	1.00	0.99	0.013
**Apr 06**	215	1003	4129	12759	0.97	0.94	1.00	0.89	0.95	0.023
**May 06**	86	387	1914	4898	0.39	0.36	0.46	0.34	0.39	0.026
**Jun 06**	19	83	334	1175	0.09	0.08	0.08	0.08	0.08	0.002
**Jul 06**	12	105	168	781	0.05	0.10	0.04	0.05	0.06	0.014

**Table 4:** Time-lagged relationship between water temperature (2 months prior to recruitment) and appearance of *Undaria pinnatifida* recruits. Field results from Brest harbour, France 2005/2006 [2].

**Field results**		**Model predictions**	
**Temperature (°C)**	**Rel. recruitment**	**Temperature (°C)**	**Rel. recruitment**
15.55556	0	8.53486	0.46355
16.8254	0	8.61046	1
16.24339	0	8.99722	0.38914
16.56085	0.109804	9.09356	0.894427
15.50265	0.054902	9.10606	1
13.38624	0.054902	9.46605	1
10.68783	0.27451	9.50739	0.36236
9.550265	0.705882	9.50883	0.4125
8.597884	1	9.56146	0.948414
8.518519	0.253394	9.75142	0.939139
10.13228	0.063348	10.0466	0.972851
12.59259	0	10.169	0.080891
15.55556	0.058824	10.2278	0.343358
16.8254	0.294118	10.3061	1
16.24339	0.294118	10.3095	0.4375
16.56085	0.352941	10.5467	0.085973
15.50265	0.176471	10.5475	1
13.38624	0.352941	10.7031	0.821991
10.68783	0.352941	10.7978	0.995475
9.550265	1	11.3494	0.912921
8.597884	0.647059	11.3703	0.077715
8.518519	0.235294	11.7769	0.075
10.13228	0.058824	11.8359	0.082369
12.59259	0	12.5988	0.040688
15.55556	0.1	12.675	0.649281
16.8254	0	12.8818	0.054299
16.24339	0.2	12.8941	0.343891
16.56085	0.3	13.1087	0.098315
15.50265	0.2	13.2682	0.36985
13.38624	0.1	13.3292	0.308791
10.68783	0.2	13.3819	0.025
9.550265	0.3	13.4321	0.054749
8.597884	1	14.9062	0.273887
8.518519	0.4	15.12	0.384615
10.13228	0.2	15.2796	0.389513
12.59259	0.11	15.3819	0.033368
15.55556	0.22291	15.5398	0.051102
16.8254	0	15.6025	0.332042
16.24339	0.055728	15.798	0.048689
16.56085	0.055728	15.8213	0.085973
15.50265	0.278638	15.9455	0.098423
13.38624	0.278638	16.0195	0.146067
10.68783	0.780186	16.2086	0.427464
9.550265	0.823529	16.3575	0.33782
8.597884	1	16.388	0.315177
8.518519	0.373702	16.6228	0.524098
10.13228	0.124567	16.8302	0.179462
12.59259	0	16.9061	0.424956
15.55556	0	17.0483	0.149321
16.8254	0.072874	17.1102	0.356742
16.24339	0.364372	17.2186	0.149317
16.56085	0.291498	17.2403	0.520599
15.50265	0.218623	17.2976	0.506787
13.38624	0.364372	18.0803	0.321267
10.68783	0.437247		
9.550265	1		
8.597884	0.615385		
8.518519	0.488688		
10.13228	0.325792		
12.59259	0		
